# Association of socioeconomic deprivation with life expectancy and all-cause mortality in Spain, 2011–2013

**DOI:** 10.1038/s41598-022-19859-1

**Published:** 2022-09-16

**Authors:** Daniel Redondo-Sánchez, María-José Sánchez, Pablo Fernández-Navarro, Bernard Rachet, Miguel Angel Luque-Fernandez

**Affiliations:** 1 Instituto de Investigación Biosanitaria Ibs.Granada, 18012 Granada, Spain; 2 Consortium for Biomedical Research in Epidemiology and Public Health (CIBER Epidemiología y Salud Pública, CIBERESP), 28029 Madrid, Spain; 3 Andalusian School of Public Health, Granada, Spain; 4 Department of Preventive Medicine and Public Health, University of Granada, Granada, Spain; 5 Cancer and Environmental Epidemiology Unit, National Centre for Epidemiology, Carlos III Institute of Health, Madrid, Spain; 6 Inequalities in Cancer Outcomes Network, Department of Non-Communicable Disease Epidemiology, London School of Hygiene and Tropical Medicine, Keppel St, London, WC1E 7HT UK

**Keywords:** Epidemiology, Public health

## Abstract

Life tables summarise a population’s mortality experience during a time period. Sex- and age-specific life tables are needed to compute various cancer survival measures. However, mortality rates vary according to socioeconomic status. We present sex- and age-specific life tables based on socioeconomic status at the census tract level in Spain during 2011–2013 that will allow estimating cancer relative survival estimates and life expectancy measures by socioeconomic status. Population and mortality data were obtained from the Spanish Statistical Office. Socioeconomic level was measured using the Spanish Deprivation Index by census tract. We produced sex- and age-specific life expectancies at birth by quintiles of deprivation, and life tables by census tract and province. Life expectancy at birth was higher among women than among men. Women and men in the most deprived census tracts in Spain lived 3.2 and 3.8 years less than their counterparts in the least deprived areas. A higher life expectancy in the northern regions of Spain was discovered. Life expectancy was higher in provincial capitals than in rural areas. We found a significant life expectancy gap and geographical variation by sex and socioeconomic status in Spain. The gap was more pronounced among men than among women. Understanding the association between life expectancy and socioeconomic status could help in developing appropriate public health programs. Furthermore, the life tables we produced are needed to estimate cancer specific survival measures by socioeconomic status. Therefore, they are important for cancer control in Spain.

## Introduction

The burden of cancer is rising globally, exerting a significant strain on populations and health systems at all income levels. Given the socio-demographic changes in Western societies, cancer control is currently one of the most complex health challenges^1^. National and regional estimates of population-based cancer survival measures serve to evaluate the effectiveness of health care systems in improving cancer outcomes^2^.

Life tables are needed to estimate the burden of cancer through several cancer survival measures using population-based cancer registry data (e.g., net survival, crude probability of death, life-years lost due to cancer, etc.)^3,4^. Net survival is an important policy-based statistic for cancer control that reflects the probability of surviving cancer in the absence of other causes of death. It is a measure that is not influenced by changes in mortality from other causes and, therefore, provides a useful measure for tracking survival across time and comparisons between socioeconomic groups or between regions^5^. In addition to being used for the estimation of cancer survival measures, life tables are the basis for calculating life expectancy (LE)^6^. LE at birth is an important indicator that reflects social inequalities in health across the life course, influenced by age, sex, geographical region, and socioeconomic status (SES). However, there are no available estimates of LE at birth by SES in Spain meaning that important cancer specific survival measures by SES for cancer control are not yet estimated in Spain.

Despite efforts worldwide and in Europe, social inequalities in cancer survival persist^7^. Social inequalities in cancer outcomes have an economic impact on health care costs^8^. Therefore, identifying and characterising socioeconomic and geographic disparities in LE can help in evaluating socioeconomic disparities in cancer survival. Identifying LE and cancer survival measures helps optimise and redistribute healthcare services more equitably. However, to estimate these cancer survival measures by SES for a specific geographical area, we need to incorporate the overall area mortality rate obtained from national or regional life tables^9^. Furthermore, life tables by socioeconomic status have not been computed in Spain yet highlighting the relevance of our work for cancer research, control, and social inequalities in health. We hypothesize that SES is associated with LE at birth, i.e., people living in more affluent areas have longer LE than people living in the most deprived areas of Spain.

Therefore, we aimed to construct life tables by sex, age, and SES at the census tract level and by provinces for the 2011–2013 period in Spain and to produce comparative estimates of LE by sex, age, region, and SES in Spain.

## Methods

### Study design, participants, data, and setting

We conducted a population-based, multilevel study to derive LE at birth by SES in Spain. Population and mortality data were drawn from the Spanish Statistical Office for the 2011–2013 period using census tract level, age, calendar year, and sex (Spanish Statistical Office data cession agreement number 67/2020). LE at birth for the 2011–2013 period is essential for cancer control and establishing temporal comparisons (i.e., trends) over time when new information from the national census will be made publicly available in Spain.

We used the layer of census tracts of the Spanish Census in 2011, with 35,960 census tracts. The mean area of the census tracts is 14.04 km^2^ (5.42mi^2^), and the mean population is 1,311 inhabitants for census tract, with the closest equivalent in the US geographic levels being the census block group^10^. To measure deprivation in each census tract, we used the Spanish Deprivation Index (SDI)^11^ created by principal component analysis^12^ using data from the Spanish 2011 census conducted by the Spanish Statistical Office (https://www.ine.es/dyngs/INEbase/en/categoria.htm?c=Estadistica_P&cid=1254735572981).

The SDI includes information from six indicators mainly related to employment and education: percentage of manual workers (employed or unemployed), percentage of occasional workers (employed or unemployed), percentage of the population with insufficient education (i.e., less than 8 years of secondary studies), and percentage of main homes without internet access^11^. While the index has no direct information about income, we discovered in a previous study that the SDI and average income per person are associated at the census tract level^13^. We used the SDI divided in quintiles (Q), where Q5 represents the 20% of census tracts which are the most deprived areas (lowest SES) and Q1 the 20% of census tracts which are the least deprived areas (highest SES).

The Internal Review Board of the Andalusian School of Public Health (CP17/00206), Granada Provincial Intern Research Review Committee and the Biomedical Ethics Committee of the Department of Health of the Andalusian Regional Government (study 0072-N-18) approved the study protocol. The study conforms to the principles embodied in the Declaration of Helsinki.

### Statistical analysis

We performed a descriptive analysis, where we first computed the crude mortality rates per 100,000 people for the overall period and by quintiles of deprivation, categories of age, and sex in Spain. To compute mortality rates, we used the total population at risk for the analysed period.

The counts of deaths and population produced by the Spanish Statistical Office were available only for five-year age groups from 0 to > 85 years (i.e. abridged). Therefore, we used a modified modelling approach described elsewhere^14^ to estimate the smoothed mortality rates using a flexible Poisson multivariable mixed-effects model^15^. Death counts were modelled in the generalised linear model framework, considering a Poisson error, using restricted cubic splines to capture the smoothed effect of age, and including the at-risk population as an offset^16^. Models were stratified according to sex. The covariates considered in the model were restricted cubic splines of age (using the mid-age of each age group), quintile of the SDI, interaction between SDI and age, and random intercept for the census tracts. The model specification is given by:

1 $$\mathrm{ln}\left(\mathrm{cases_{age,SDI}}/\mathrm{population_{age,SDI}}\right)\mathrm{_j }=\upbeta _0 +\mathrm{ f}_{1}\left(\mathrm{age}\right)+\sum_{\mathrm{i}=2}^{5}{\upbeta }_{\mathrm{i}}\times \mathrm{ Quintile\, {SDI}}+\mathrm{f}_{2}(\mathrm{quintile \, SDI_i }\times \mathrm{ age}) +\mathrm{ Q_j}$$

where Q_**j**_ is the random intercept for the j census tract, and f_1_ and f_2_ represent the restricted cubic splines for age and the interaction between age and the quintiles of SDI, respectively.

We used a data-adaptive cross-validated approach to identify the best position and number of knots for the mean-centred age of 60 years. The knot positions were fixed at ages 2, 12, 22, 32, 42, 52, 67, and 82 based on the lowest cross-validated mean absolute error of a set of models with different knot positions^17^.

We included the census tract as a random intercept [Q_j_ in (Eq. 1)] to account for the non-spatial variability and improve the model fit, given that the random effects approach penalised smoothing splines, which reduced overfitting^18^. From the fitted model, we predicted the smoothed mortality rates accounting for the random intercept by age group and quintile of deprivation, stratified by sex. We derived LE for people ≥ 75 years old by census tracts from the life tables and presented it in choropleth maps. LE quintiles were computed by dividing the census tracts in five groups, where Q1 represents the 20% of census tracts which have the lowest LE, and Q5 the 20% of census tracts which have the highest LE. Furthermore, we computed the LEs at birth by province, by weighting the LE for all the census tracts of the province with the population size of each census tract.

To assess the goodness-of-fit of the model, we compared the predicted and observed mortality rates. We derived 95% confidence intervals for the mortality rates using the Delta method^19^.

We used Stata v.16.1 (StataCorp, College Station, Texas, USA) and R v.4.1.0 (R Foundation for Statistical Computing, Vienna, Austria) for statistical analysis and mapping. The syntax files for the computation of the life tables and the derived life tables are available on GitHub (https://github.com/migariane/Spanish_LifeTablesByDeprivation).

### Ethics approval and consent to participate

The Internal Review Board of the Andalusian School of Public Health (CP17/00206), Granada Provincial Intern Research Review Committee and the Biomedical Ethics Committee of the Department of Health of the Andalusian Regional Government (study 0072-N-18) approved the study protocol. The study conforms to the principles embodied in the Declaration of Helsinki.

## Results

**Figure 1:**
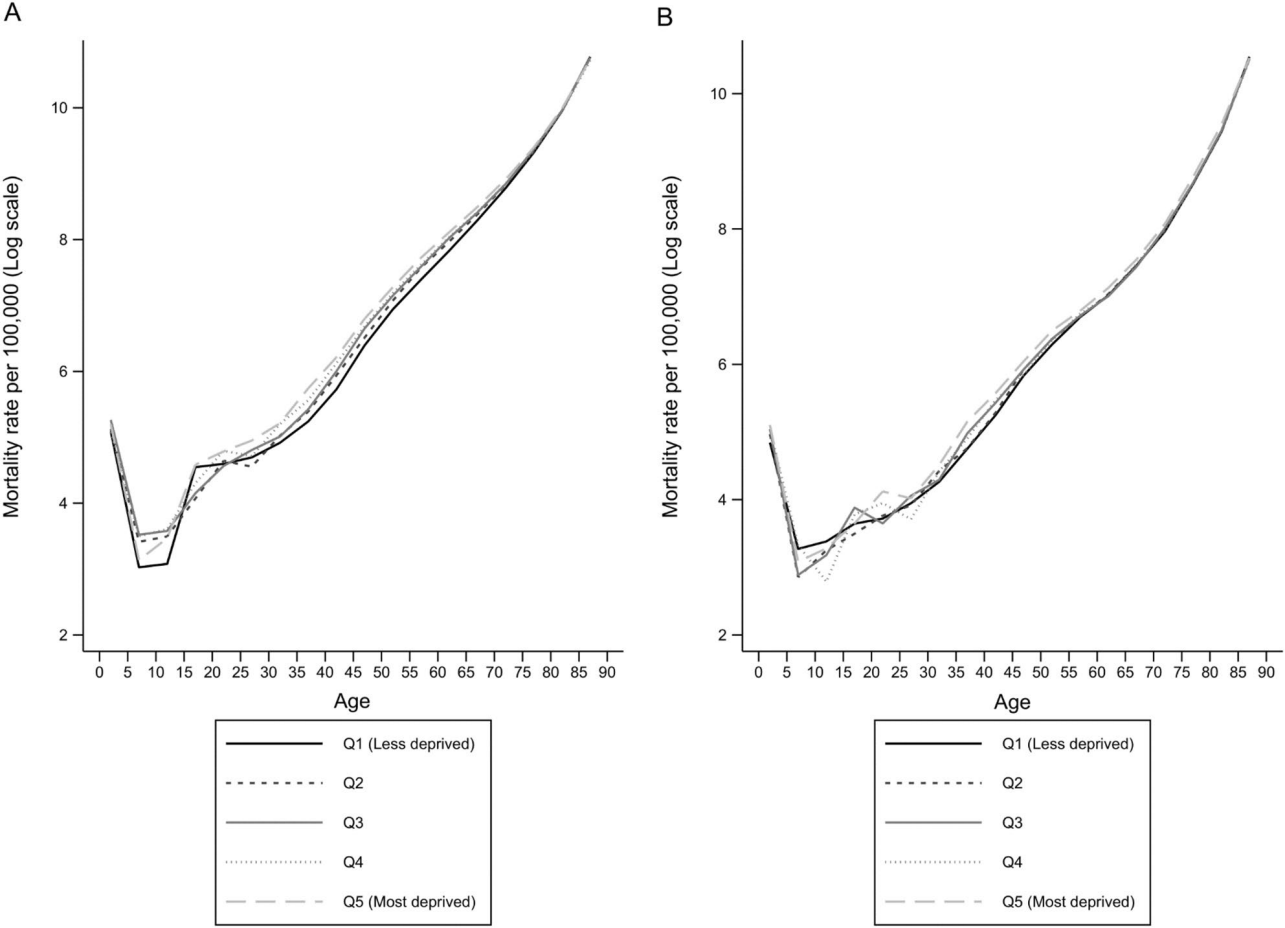
Observed mortality rates per 100,000 people by deprivation quintile, age group and sex (**A**: men, **B**: women) in Spain, 2011–2013.

Overall, the mortality rates were higher among men than among women and also higher among people living in the most deprived areas of Spain (Table 1). Mortality rates remained stable for men and women over the 2011–2013 period (i.e. p-value of trend > 0.05). Standardised mortality rates using the indirect method^20^ were approximately 20% and 10% higher among men and women living in the most deprived areas, respectively, than among those living in the least deprived areas. The observed mortality rates over the 2011–2013 period in Spain by age group and quintiles of deprivation stratified by sex showed a u-shape with higher mortality rates at the extremes of the age distribution (Fig. 1). Supplementary Fig. 1 shows the goodness-of-fit of the model. The concordance of the observed vs. fitted mortality rates by age and quintiles of deprivation was good among all quintiles for > 20 years, with small discrepancies at earlier ages. Overall, the mortality rates among people living in the most deprived areas were higher among those aged > 15 years for both men and women. The mortality gap due to deprivation disappeared at older ages (i.e. ≥ 75 years) and was not evident at younger ages (i.e. < 15 years). Biggest differences in mortality rates were observed between 30 and 70 years of age (Fig. 1).

**Table 1:** Deaths, population, mortality rate, and standardized mortality rate with 95% confidence interval by sex, year and deprivation quintiles in Spain between 2011 to 2013.

Year	Quintile of SDI	Deaths	Population	Mortality rate per 10^5^ people (95% CI)	SMR (95% CI)
**Males**					
2011	Q1	35,217	5,256,845	6.69 (6.63–6.77)	Ref
	Q2	39,405	5,073,220	7.77 (7.69–7.84)	1.05 (1.03–1.08)
	Q3	41,134	4,759,007	8.64 (8.56–8.72)	1.09 (1.07–1.11)
	Q4	39,745	4,270,710	9.31 (9.21–9.40)	1.10 (1.08–1.12)
	Q5	39,523	3,921,491	10.08 (9.98–10.18)	1.17 (1.15–1.19)
2012	Q1	36,126	5,288,462	6.83 (6.76–6.90)	Ref
	Q2	40,656	5,075,266	8.01 (7.93–8.08)	1.06 (1.04–1.08)
	Q3	42,394	4,750,182	8.92 (8.84–9.01)	1.10 (1.08–1.12)
	Q4	41,073	4,254,020	9.65 (9.56–9.75)	1.12 (1.10–1.14)
	Q5	40,949	3,888,492	10.53 (10.43–10.63)	1.20 (1.18–1.22)
2013	Q1	35,551	5,272,262	6.74 (6.73–6.81)	Ref
	Q2	39,766	5,039,926	7.89 (7.81–7.97)	1.06 (1.05–1.08)
	Q3	40,643	4,703,703	8.64 (8.56–8.75)	1.08 (1.07–1.10)
	Q4	39,752	4,194,731	9.48 (9.38–9.57)	1.12 (1.10–1.14)
	Q5	39,314	3,832,194	10.26 (10.16–10.36)	1.19 (1.17–1.21)
**Females**					
2011	Q1	38,283	5,657,980	6.77 (6.69–6.83)	Ref
	Q2	37,608	5,270,843	7.14 (7.06–7.21)	0.98 (0.96–0.99)
	Q3	37,515	4,853,180	7.13 (7.06–7.21)	0.99 (0.98–1.00)
	Q4	35,979	4,277,989	8.41 (8.32–8.50)	1.01 (0.99–1.03)
	Q5	34,734	3,845,162	9.03 (8.94–9.13)	1.06 (1.04–1.07)
2012	Q1	39,696	5,699,851	6.96 (6.90–7.03)	Ref
	Q2	39,340	5,286,365	7.44 (7.37–7.52)	0.99 (0.98–1.00)
	Q3	39,459	4,854,909	8.13 (8.04–8.86)	1.01 (0.99–1.03)
	Q4	37,451	4,268,555	8.77 (8.68–8.86)	1.02 (1.01–1.04)
	Q5	37,020	3,815,310	9.70 (9.60–9.80)	1.10 (1.08–1.12)
2013	Q1	38,846	5,691,572	6.83 (6.76–6.89)	Ref
	Q2	38,137	5,266,307	7.24 (7.20–7.31)	0.98 (0.97–0.99)
	Q3	38,004	4,824,427	7.88 (7.79–7.96)	1.00 (0.98–1.01)
	Q4	36,221	4,224,892	8.57 (8.48–8.66)	1.02 (1.00–1.04)
	Q5	35,242	3,772,367	9.34 (9.25–9.44)	1.08 (1.06–1.10)

*SDI* Spanish Deprivation Index, *Q* deprivation quintiles, *CI* confidence interval, *SMR* standardized mortality rate (indirect method).

**Figure 2:**
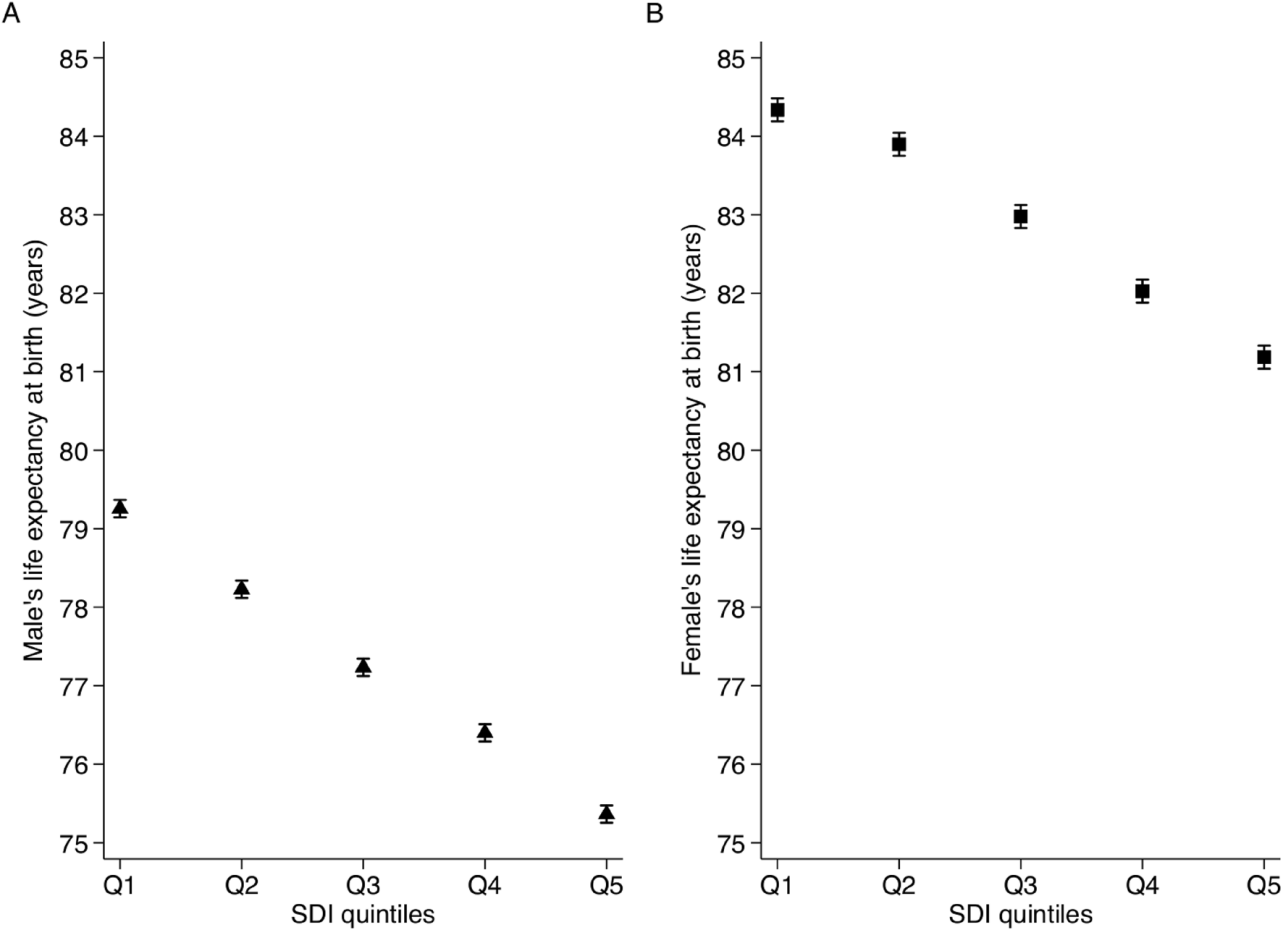
Life expectancy at birth (in years) by quintiles of deprivation and sex (**A**: men, **B**: women) in Spain, 2011–2013.

There was a consistent gap in LE at birth between categories of sex and deprivation in Spain during the 2011–2013 period (Fig. 2A: men, Fig. 2B: women). LE at birth for the 2011–2013 period for women was 5.6 years higher than that for men (i.e. females LE at birth: 82.9 years vs. males LE at birth: 77.3). Regarding deprivation, women living in the least deprived census tracts lived 3.2 more years than women living in the most deprived areas (i.e., LE at birth of 84.3 vs. 81.2 years). Furthermore, men living in the least deprived areas in Spain lived for 3.8 more years than men living in the most deprived areas (i.e., LE at birth of 79.2 vs. 75.4 years). There was evidence of a decreasing linear trend of LE at birth across levels of deprivation i.e., from Q1 (the least deprived quintile) to Q5 (the most deprived quintile) for both men and women (p-value of trend < 0.01).

**Figure 3:**
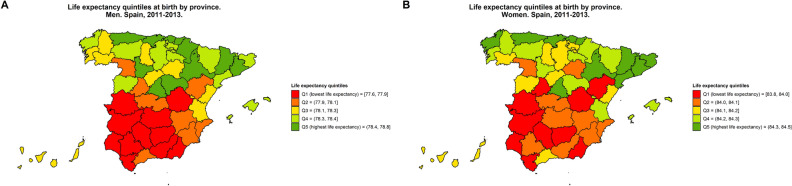
Life expectancy quintiles at birth by province and sex (**A**: men, **B**: women) in Spain, 2011–2013.

The quintiles of the LE at birth by province (Fig. 3) showed a clear geographical distribution, with the majority of the provinces with shorter LE (i.e., LE Q1) for both men (Fig. 3A) and women (Fig. 3B) being located in the southwest of Spain.

**Figure 4:**
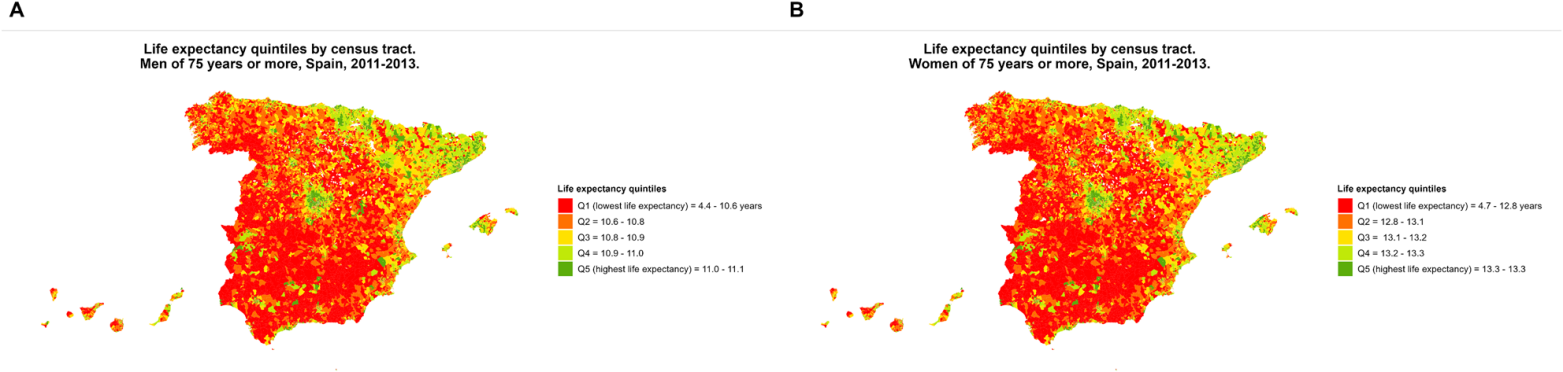
Life expectancy quintiles among people aged ≥ 75 years old by census tract and sex (**A**: men, **B**: women) in Spain, 2011–2013.

Figure 4 shows the quintiles of LE among people aged ≥ 75 years by census tract in Spain from 2011 to 2013. The map shows a singular pattern characterised by a shorter LE among those aged ≥ 75 years in rural areas, that usually have larger census tracts than urban areas. On the contrary, higher LE is more frequent at census tracts in provincial capitals, and therefore highly industrialised areas, such as Madrid and Catalonia, for both sexes.

## Discussion

Overall, we found a consistent LE gap at birth according to SES for both sexes in Spain during the 2011–2013 period. However, the gap was wider among men than among women, with the least deprived male group experiencing shorter LE at birth than the most deprived female group. Furthermore, we found a geographical pattern characterised by shorter LE at birth in the southwest for both sexes in Spain.

Consistent with our results, a previous study showed trends in socioeconomic inequalities in all-cause mortality in small areas of 33 Spanish cities for both sexes, but these inequalities were greater in men than in women^21^. Overall LE at birth and among people aged ≥ 75 years in our study was consistent with official national and international statistics^22^. The north-to-south pattern discovered, with higher LE in the northern regions (which also have higher SES), is well-documented from another study, where higher mortality rates were associated with particular geographical areas in Spain^23^. Furthermore, our results on LE at birth by deprivation are consistent with those of other European studies^24^. In the UK, LE at birth in 2005 presented a similar pattern, with the highest LE in the most affluent groups compared to the most deprived group, with a gap between 2.7 and 5.0 years for males and between 2.5 and 3.6 years for females^25^. Overall, differences in LE are seen by deprivation and by region, and the regions with higher LE are also the least deprived^25^. Similar to the geographical pattern found in Spain, a regional pattern characterised by a clear north–south gradient was found in the UK, with deprivation explaining most of the geographical variation in LE^25^. A similar pattern was found in Portugal, where LE at birth for the 2010–2012 period was higher among the least deprived people^26^. However, differences between the most and least deprived people were about 2.0 years higher for men and 1.0 year higher for women living in the least deprived areas of Portugal^26^. Consistent with the findings in both Spain and the UK, LE differences progressively disappeared with increasing age in Portugal^26^. Similar findings related to the association between shorter LE and lower income or education have been reported in other European countries, including Norway, Denmark, and Belgium^27–29^^.^

In the USA and Canada, there is evidence supporting higher LE among people with higher income between 2002 to 2014, and geographical variation in LE was correlated with the characteristics of local areas in terms of health behaviour^30,31^.

### Strengths

Our study provided evidence of an LE gap between socioeconomic groups. Furthermore, the results of the study are consistent with those of other international and European studies regarding LE by deprivation. However, our study provides more and updated evidence regarding the presence of an LE gap by sex and region. To the best of our knowledge, this is the first study to assess LE at birth by age, sex, and SES in Spain. Our findings are relevant and important for the development of public health policies tackling social inequalities in health targeting the LE gap by socioeconomic status (i.e., people from more affluent regions live longer than people living in the most deprived areas). Furthermore, the life tables by province we have produced and made freely available on the GitHub repository are essential for the computation of cancer-specific survival estimates adjusted for SES. The use of the life tables in population-based cancer research can help foster novel evidence on cancer survival inequalities by SES in Spain.

### Limitations

In our study, the SDI we referred to was of 2011, while mortality was measured in the 2011–2013 period. This is because the SDI is based on the national census data of Spain, which are generated every 10 years. However, it is the first time LE by SES has been computed and the life tables by age, sex, and SES that we produced will serve as a baseline reference for monitoring trends during the coming years and when the new Spanish census result are made available. We argue that the effect of deprivation on mortality will be consistent over time, but trends and changes over time must be monitored. Furthermore, the abridged nature of the data (i.e., aggregated data on age) reduces the possibility of identifying mortality differences across age groups. However, we attempted to accommodate this using a flexible modelling framework.

Another limitation was the layer of census tracts undergoing continuous changes over time. However, for our analysis, we used the fixed layer of census tracts of the National Census of 2011 (1st November 2011), which was the same as that used for the SDI, except for mortality and population data (i.e., the mortality data used the most up-to-date census tract layer at the date of each death and the population data used the census tract layer on 1^st^ January of each year). In sensitivity analysis, the linkage results between population figures, mortality data, and SDI are shown in Supplementary Table 1. Overall, the unlinked deaths (mortality-population link) were around 1.7%, with the average number of deaths lost per year being 6567 out of 391,913 (Supplementary Table 1). The unlinked census tracts for the population-SDI link were below 0.6% for all years, with an average of 90 census tracts lost for each sex and year out of an average of 36,003 census tracts per sex and year (Supplementary Table 2). Finally, we assumed that the census tract of residence at death was the same throughout the life of a person.

## Conclusions

In summary, we found an important LE gap according to sex and SES in Spain during the 2011–2013 period. The LE gap was more pronounced among men than among women and less pronounced among people aged < 75 years. Understanding the factors explaining the association between LE and SES could be helpful in the development of appropriate public health programs. The generated life tables, made available for researchers on the GitHub repository: https://github.com/migariane/Spanish_LifeTablesByDeprivation, are an essential statistical tool for estimating the net cancer-specific survival by SES, a useful indicator for detecting socioeconomic inequalities in cancer survival.

## Supplementary Information

Supplementary Figure 1.

Supplementary Table 1.

Supplementary Table 2.

## Data Availability

The data that support the findings of this study are available from the Spanish Statistical Office (INE). The syntax files for the computation of the life tables and the derived life tables are available on GitHub (https://github.com/migariane/Spanish_LifeTablesByDeprivation).

## References

[1] Mander T (2014). Longevity and healthy ageing—Will healthcare be drowned by the grey tsunami or sunk by the demographic iceberg?. Post Reprod. Health.

[2] Allemani, C., Matsuda, T., Di Carlo, V. *et al.* Global surveillance of trends in cancer survival 2000–14 (CONCORD-3): Analysis of individual records for 37 513 025 patients diagnosed with one of 18 cancers from 322 population-based registries in 71 countries. *Lancet* (2018). 10.1016/S0140-6736(17)33326-3 PMC5879496 29395269

[3] Belot A (2019). Summarizing and communicating on survival data according to the audience: A tutorial on different measures illustrated with population-based cancer registry data. Clin. Epidemiol..

[4] PoharPerme M, Estève J, Rachet B (2016). Analysing population-based cancer survival—Settling the controversies. BMC Cancer.

[5] Syriopoulou E, Morris E, Finan PJ, Lambert PC, Rutherford MJ (2019). Understanding the impact of socioeconomic differences in colorectal cancer survival: Potential gain in life-years. Br. J. Cancer.

[6] Arias E, Curtin LR, Wei R, Anderson RNUS (2008). decennial life tables for 1999–2001, United States life tables. Natl Vital Stat. Rep..

[7] Hashim D, Erdmann F, Zeeb H (2019). Editorial: Social inequities in cancer. Front. Oncol..

[8] Luengo-Fernandez R, Leal J, Gray A, Sullivan R (2013). Economic burden of cancer across the European Union: A population-based cost analysis. Lancet Oncol..

[9] Mariotto AB (2014). Cancer survival: An overview of measures, uses, and interpretation. J. Natl Cancer Inst. Monogr..

[10] Krieger N (2002). Geocoding and monitoring of US socioeconomic inequalities in mortality and cancer incidence: Does the choice of area-based measure and geographic level matter? The Public Health Disparities Geocoding Project. Am. J. Epidemiol..

[11] Duque I (2021). Índice de privación en España por sección censal en 2011. Gac. sanit..

[12] Allik M (2020). Creating small-area deprivation indices: A guide for stages and options. J. Epidemiol. Commun. Health.

[13] Redondo-Sánchez D (2021). Lung, breast and colorectal cancer incidence by socioeconomic status in Spain: A population-based multilevel study. Cancers (Basel).

[14] Rachet B (2015). Multivariable flexible modelling for estimating complete, smoothed life tables for sub-national populations. BMC Public Health.

[15] Luque-Fernandez MA (2016). Adjusting for overdispersion in piecewise exponential regression models to estimate excess mortality rate in population-based research. BMC Med. Res. Methodol..

[16] Faraway J (2006). Extending the Linear Model with R Generalized Linear, Mixed Effects and Nonparametric Regression Models.

[17] Bates, S., Hastie, T. & Tibshirani, R. Cross-validation: What does it estimate and how well does it do it? http://arxiv.org/abs/2104.00673 (published online first: 1 Apr 2021). 10.1080/01621459.2023.2197686 PMC11412612 39308484

[18] Nielsen JD, Dean CB (2008). Clustered mixed nonhomogeneous Poisson process spline models for the analysis of recurrent event panel data. Biometrics.

[19] Delta method in epidemiology: An applied and reproducible tutorial. https://migariane.github.io/DeltaMethodEpiTutorial.nb.html.

[20] Lash TL, VanderWeele TJ, Haneuse S, Rothman KJ (2020). Modern Epidemiology.

[21] Marí-Dell’Olmo M, Gotsens M, Palència L (2016). Trends in socioeconomic inequalities in mortality in small areas of 33 Spanish cities. BMC Public Health.

[22] Mortality and life expectancy statistics—Statistics explained. https://ec.europa.eu/eurostat/statistics-explained/index.pAuthor?title=Mortality_and_life_expectancy_statistics.

[23] Regidor E (2015). The association of geographic coordinates with mortality in people with lower and higher education and with mortality inequalities in Spain. PLoS ONE.

[24] Welsh CE, Matthews FE, Jagger C (2021). Trends in life expectancy and healthy life years at birth and age 65 in the UK 2008–2016, and other countries of the EU28: An observational cross-sectional study. Lancet Reg. Health Eur..

[25] Woods LM (2005). Geographical variation in life expectancy at birth in England and Wales is largely explained by deprivation. J. Epidemiol. Commun. Heal..

[26] Antunes L, Mendonça D, Ribeiro AI, Maringe C, Rachet B (2019). Deprivation-specific life tables using multivariable flexible modelling—Trends from 2000–2002 to 2010–2012, Portugal. BMC Public Health.

[27] Renard F, Devleesschauwer B, Van Oyen H (2019). Evolution of educational inequalities in life and health expectancies at 25 years in Belgium between 2001 and 2011: A census-based study. Arch. Public Health.

[28] Brønnum-Hansen H, Baadsgaard M (2012). Widening social inequality in life expectancy in Denmark. A register-based study on social composition and mortality trends for the Danish population. BMC Public Health.

[29] Kingle JM, Minet JH, Øverland S (2019). Association of household income with life expectancy and cause-specific mortality in Norway, 2005–2015. JAMA.

[30] Chetty R (2016). The association between income and life expectancy in the United States, 2001–2014: Association between income and life expectancy in the United States. JAMA.

[31] Bushnik T, Tjepkema M, Martel L (2020). Socioeconomic disparities in life and health expectancy among the household population in Canada. Heal. Rep..

