# Supplementary material for: Association of socioeconomic deprivation with life expectancy and all-cause mortality in Spain, 2011–2013
Source: Sci Rep. 2022 Sep 16;12:15554. doi: 10.1038/s41598-022-19859-1 (PMC9481591; doi:10.1038/s41598-022-19859-1)

Supplementary Figure 1. Goodness-of-fit of the model by sex (A: men, B: women), quintile of deprivation, and age group.

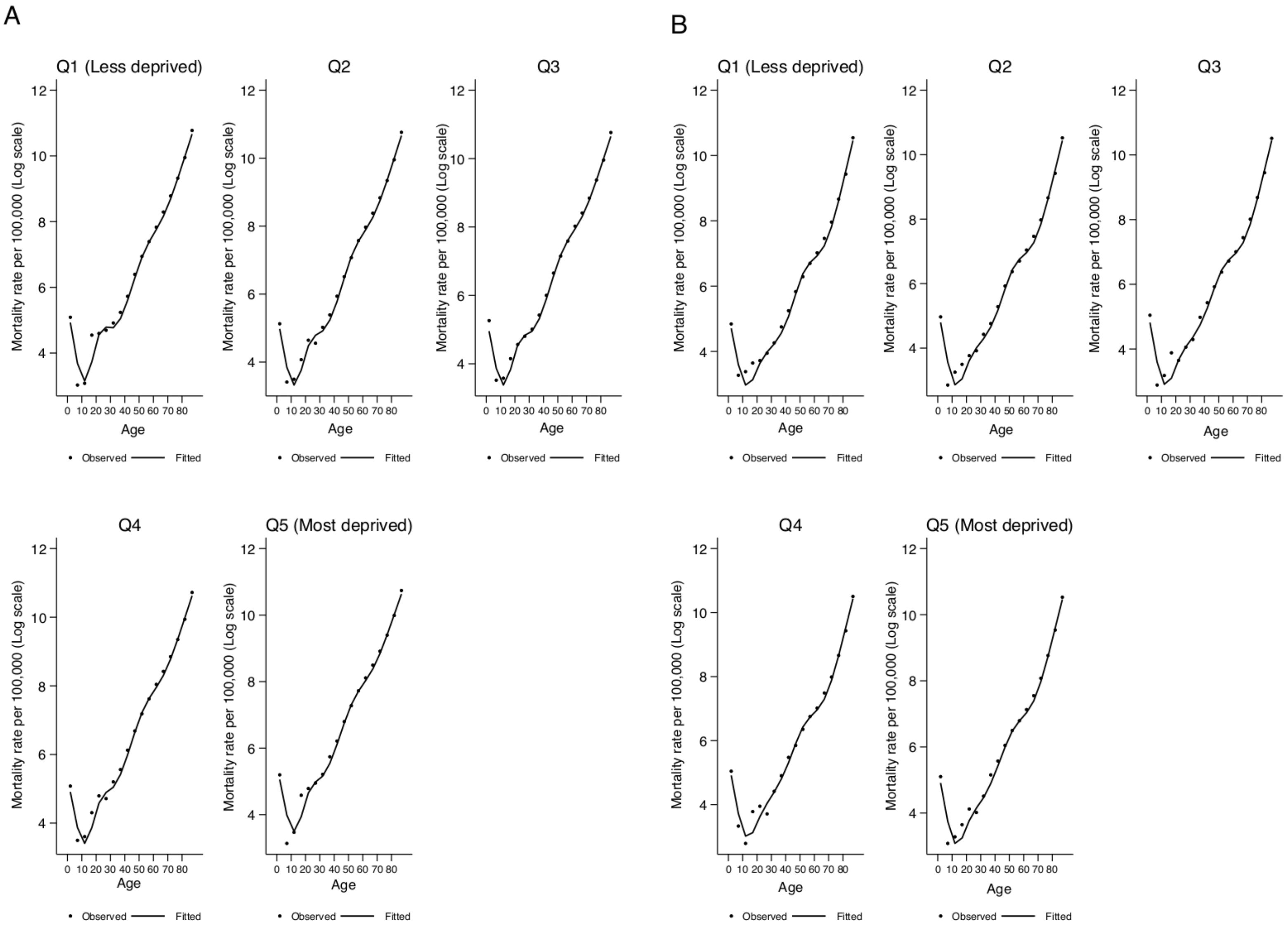

Supplement: Supplementary file 1 — Supplementary Figure 1. [file 41598_2022_19859_MOESM1_ESM.pdf]
