# Supplementary material for: Association of socioeconomic deprivation with life expectancy and all-cause mortality in Spain, 2011–2013
Source: Sci Rep. 2022 Sep 16;12:15554. doi: 10.1038/s41598-022-19859-1 (PMC9481591; doi:10.1038/s41598-022-19859-1)
Supplement: Supplementary file 2 — Supplementary Table 1. [file 41598_2022_19859_MOESM2_ESM.docx]

**Supplementary Table 1.** Mean population by census tract and number of deaths available for analysis after linkage of mortality and population data in Spain 2011-2013.

| **Year** | **Mean population by census tract** | **Total deaths** | **Linked deaths** | **Unlinked deaths** | **% unlinked deaths** |
| --- | --- | --- | --- | --- | --- |
| 2011 | 1,312 | 386,017 | 379,165 | 6,852 | 1.8% |
| 2012 | 1,314 | 401,122 | 394,485 | 6,637 | 1.7% |
| 2013 | 1,307 | 388,600 | 382,388 | 6,212 | 1.6% |
