# Supplementary material for: Association of socioeconomic deprivation with life expectancy and all-cause mortality in Spain, 2011–2013
Source: Sci Rep. 2022 Sep 16;12:15554. doi: 10.1038/s41598-022-19859-1 (PMC9481591; doi:10.1038/s41598-022-19859-1)
Supplement: Supplementary file 3 — Supplementary Table 2. [file 41598_2022_19859_MOESM3_ESM.docx]

**Suppl. Table 2.** Number of census tracts lost and available for analysis after linkage with mortality and deprivation data by sex

|  |  |  | **Census tracts lost** | |
| --- | --- | --- | --- | --- |
| **Year** | **Total Census tracts** | **Linked census tracts** | **n** | **%** |
| **Males** | | | | |
| 2011 | 35,960 | 35,957 | 3 | 0.01% |
| 2012 | 35,978 | 35,868 | 110 | 0.31% |
| 2013 | 36,071 | 35,915 | 156 | 0.43% |
| **Females** | | | | |
| 2011 | 35,960 | 35,957 | 3 | 0.01% |
| 2012 | 35,978 | 35,914 | 64 | 0.18% |
| 2013 | 36,071 | 35,867 | 204 | 0.57% |
|  |  |  |  |  |
|  |  |  |  |  |
|  |  |  |  |  |
|  |  |  |  |  |
